# Acute branch retinal arteriolar occlusion after intravascular ultrasound: a case report

**DOI:** 10.3389/fcvm.2025.1543400

**Published:** 2025-02-20

**Authors:** Gaixia Zhai, Chao Sun, Hongliang Tian, Yuanzhen Su

**Affiliations:** ^1^ Department of Ophthalmology, Zibo Central Hospital, Zibo, Shandong, China; ^2^ Department of Cardiology, Zibo Central Hospital, Zibo, Shandong, China

**Keywords:** branch retinal arteriolar occlusion, intravascular ultrasound, case report, coronary heart disease, acute

## Abstract

Intravascular ultrasound is mainly used for the diagnosis and interventional treatment of coronary heart disease. Retinal artery occlusion caused by intravascular ultrasound is very rare. We report a case of acute branch retinal arteriolar occlusion after intravascular ultrasound examination of the coronary artery in a patient with coronary heart disease. A 79-year-old male patient diagnosed with coronary heart disease suddenly complained of a black shadow blocking his left eye approximately 3 min after the intravascular ultrasound examination was completed. The patient was diagnosed with branch retinal arteriolar occlusion in the left eye after completing ophthalmic examination. To the best of our knowledge, this is the first report of acute branch retinal arteriolar occlusion after a coronary artery intravascular ultrasound examination.

## Introduction

The central retinal artery and its branch arterioles are considered terminal branches providing blood to the central retina. Disruption of blood flow leads to ischemia of the inner retinal cell layers and ganglion cell death, characterized by sudden and painless visual impairment. Retinal artery occlusion may affect the main central retinal artery or its arteriolar branches with varying degree of visual impairment dependent upon the extent of ischemic damage to the central retinal. Many different causes of retinal artery occlusion have been identified with proximal embolism being considered one of the most important pathophysiologic mechanisms.

Coronary heart disease is a common heart disease that leads to myocardial ischemia, hypoxia, and even necrosis via various mechanisms, and it has high morbidity and mortality rates worldwide (1, 2). We report a case of branch retinal arteriolar occlusion after intravascular ultrasound examination of the coronary artery in a patient with coronary heart disease.

## Case report

A 79-year-old male with known coronary heart disease and past coronary artery stenting of the left anterior descending artery presented to our hospital with new chest tightness and discomfort, which developed during the last month. Coronary CT angiography performed in our outpatient department showed that the left anterior descending coronary stent was unobstructed, and the right coronary artery had moderate stenosis in the proximal segment and severe stenosis in the middle segment. We planned to review the coronary angiography and admit the patient for coronary heart disease. The patient has a history of hypertension of 3 months duration, with a maximum blood pressure of 160/70 mmHg, and is currently not being administered antihypertensive medication. In addition, the patient had high fasting blood sugar levels for 3 months but denied any history of eye or other diseases. Upon admission, no significant abnormalities were found in the heart and lungs during physical examination. The three myocardial markers and N-terminal B-type natriuretic peptide precursor tests showed no significant abnormalities. The electrocardiogram showed no significant abnormalities, but the chest CT scan showed small nodules in both lungs and calcified plaques in the coronary and aortic arteries. Cardiac ultrasound revealed left atrial enlargement, aortic valve calcification, and mild reflux. The diagnoses upon admission were (1) acute coronary syndrome, (2) coronary atherosclerotic heart disease after intracoronary stent implantation, (3) grade 2 hypertension (extremely high risk), and (4) impaired glucose tolerance.

**Figure 1:**
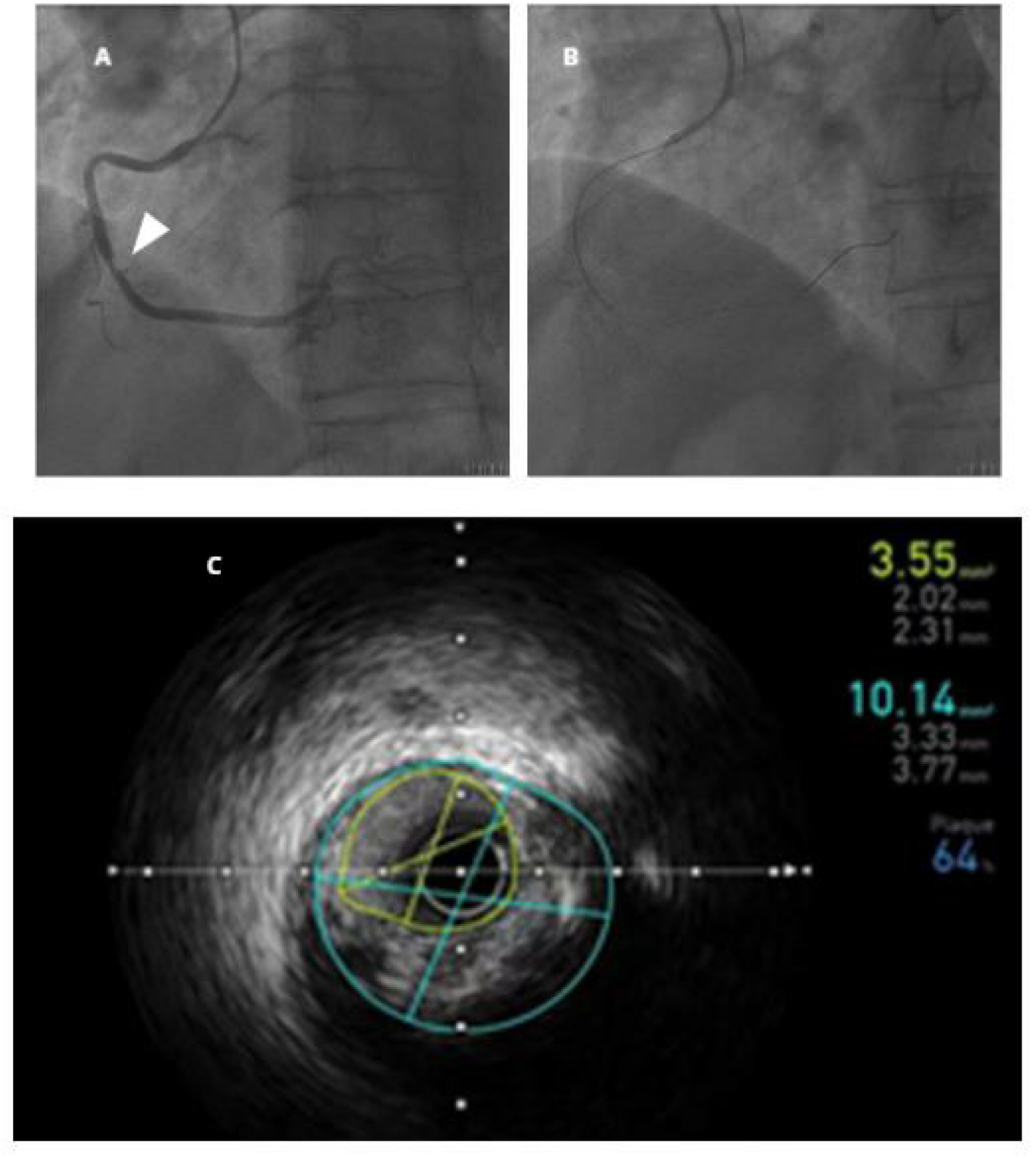
**(A)** Right coronary angiography, with white arrows indicating the location of vascular lesions; **(B,C)** intravascular ultrasound examination.

Before the operation, the patient was given aspirin and clopidogrel bisulfate for antiplatelet therapy and low-molecular-weight heparin calcium for anticoagulation. On the second day of admission, a coronary angiography was performed in the cardiac catheterization room. Heparin anticoagulation therapy was administered to the patient during the operation. The angiographic report showed that the anterior descending branch had a stent shadow in the proximal segment, no restenosis in the stent, and approximately 30%–40% stenosis in the middle segment; the circumflex branch had approximately 30%–40% proximal stenosis; and the right coronary artery had approximately 50%–60% stenosis in the proximal segment and approximately 70% localized stenosis in the middle segment (Figure 1).

**Figure 2:**
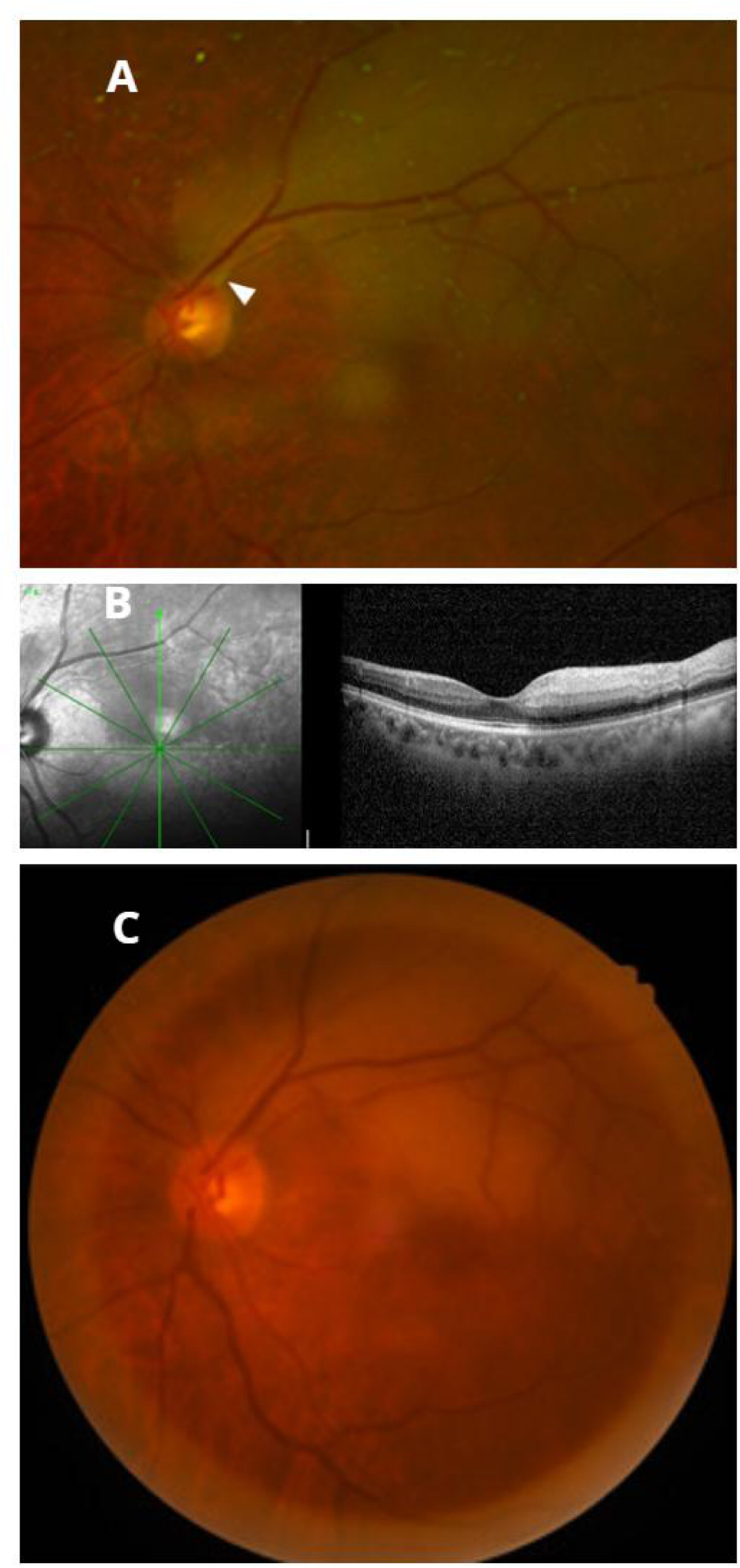
**(A)** Optos ultra-widefield imaging system: the white pointed tip indicates the location of the gray-white thrombus in the left temporal branch of the retinal artery. **(B)** Optical coherence tomography shows edema, thickening, and enhanced reflex in the inner layer of the temporal retina of the left eye. **(C)** Photograph of the posterior pole of the fundus: the retinal artery of the superior temporal branch became thinner with gray edema of the superior temporal retina.

After coronary angiography, the patient did not report any discomfort in the eyes or other parts of the body. After the patient and their family consented, intravascular ultrasound examination was performed on the right coronary artery (Figure 1). After the SAL.75 guide tube was in place, the Sion guidewire was placed in the distal segment of the right coronary artery. The intravascular ultrasound examination showed that the minimum cross-sectional area of the right coronary artery was approximately 3.55 mm^2^, with a plaque burden of 64%. Therefore, coronary intervention therapy was proposed. At this point, approximately 3 min after the intravascular ultrasound examination the patient complained of a black shadow blocking his left eye. Considering the possibility of left fundus hemorrhage in the patient, the coronary intervention treatment was temporarily suspended, and the surgery was terminated after consultation with the patient's family. We immediately consulted with an ophthalmologist. The ophthalmologist diagnosed branch retinal arteriolar occlusion in the left eye after bedside direct fundoscopy. The necessity and risks of intravenous thrombolysis for retinal artery occlusion were explained to the patient and their family. After careful consideration, the patient and their family refused intravenous thrombolysis treatment. Anterior chamber puncture, eyeball massage to reduce intraocular pressure, sublingual administration of nitroglycerin, retrobulbar injection of atropine to dilate blood vessels, and systemic vasodilator administration were performed. Because of the nighttime emergency, no objective fundus examination could be performed. A detailed ophthalmologic examination was performed in the special examination room of the ophthalmology clinic the morning after the onset of the disease. Ophthalmological examination of the left eye revealed that the visual acuity (Log MAR) was 0.3, the intraocular pressure was 15 mmHg, the conjunctiva was free of congestion, the cornea was transparent, the pupil was round, the direct light reflection was dull, the lens was turbid, and the retinal artery of the superior temporal branch became thinner, with gray edema of the superior temporal retina. Fundus photos and optical coherence tomography are shown in Figure 2. Therefore, the patient was diagnosed with left eye branch retinal arteriolar occlusion and left eye senile cataract.

The cranial MRI did not show any new cerebral infarction. Carotid artery ultrasound revealed a thickening of the intima-media and multiple plaques in both carotid arteries. After 1 week of treatment, the patient reported no significant improvement in the occlusion of the left eye by the black shadow. The patient and their family requested to be discharged. At discharge the diagnoses were: (1) coronary atherosclerotic heart disease with unstable angina pectoris after coronary stent implantation, (2) grade 2 hypertension (extremely high risk), (3) impaired glucose tolerance, (4) left eye branch retinal arteriolar occlusion, and (5) left eye senile cataract.

## Discussion

Retinal artery occlusion is considered an ophthalmological emergency, the onset of which can lead to a sudden decline or even loss of vision. The causes of retinal artery occlusion are complex and mainly involve arterial spasm, embolism, endarteritis or atherosclerosis, and other causes of retinal artery blood flow interruption, resulting in retinal tissue hypoxia, degeneration, and necrosis. Common emboli include cholesterol emboli, platelet fibrin emboli, and calcification emboli. Cholesterol emboli are the most common, and they are relatively small and reflect yellow light. Platelet fibrin emboli are common in cases of atherosclerotic plaques, and they are large and gray-white in color. Calcified emboli are relatively rare, mostly single, white in color, dull, and oval in shape.

Retinal artery occlusion caused by coronary angiography and coronary intervention is very rare. Some scholars have reported retinal artery occlusion after a percutaneous coronary intervention (3–5). Previous studies have reported several cases of retinal artery occlusion related to coronary angiography (6–8). Branch retinal arteriolar occlusion occurred approximately 30 min after the completion of coronary angiography in this patient and only approximately 3 min after the completion of intravascular ultrasound examination. In addition, the patient had not undergone the interventional treatment when the retinal artery occlusion occurred. There was a close relationship between the intravascular ultrasound examination and the branch retinal arteriolar occlusion. To our knowledge, there are currently no reports of retinal artery occlusion after a coronary artery intravascular ultrasound examination.

The patient in this case developed branch retinal arteriolar occlusion shortly after intravascular ultrasound examination, which is likely due to the shedding or tearing of the intima or the detachment of small coronary artery plaques caused by the entry and exit of the intravascular ultrasound catheter used during the ultrasound examination. Small plaques may also exist in blood vessels in other parts of the body, but there may be no obvious symptoms in other body parts because of compensatory blood flow via collateral circulation. However, the retinal artery is a terminal artery, and once it is blocked, patients may experience significant visual impairment or visual field defects. The patient developed branch retinal arteriolar occlusion 3 min after the procedure, possibly due to tiny emboli dislodged by the ultrasound catheter that circulated to the retinal branch artery after passing through several blood channels in the body. The diameter of the branch retinal artery is very small, making it more likely to be occluded. In addition, we observed that the ultrasound catheter probably touched the plaque during the ultrasound examination. Moreover, the brightness and morphology of the thrombus during the fundus examination were highly homologous to the echo intensity of the plaque in contact with the catheter during the intravascular ultrasound examination. Therefore, we concluded that the thrombus in the retinal branch artery of the patient was closely related to the intravascular ultrasound examination. To the best of our knowledge, this is the first report of acute branch retinal arteriolar occlusion after a coronary artery intravascular ultrasound examination.

It is important to note, that conservative treatment approaches in retinal artery occlusion, such as ocular massage or anterior chamber paracentesis have not been shown to be effective in randomized controlled trials. We applied the aforementioned measures because intravenous thrombolysis treatment was refused by the patient and their family. Recent studies report a favorable visual outcome in patients with retinal artery occlusion receiving early intravenous thrombolysis—however randomized controlled trials are still ongoing (e.g., the REVISION trial) (9).

Retinal artery occlusion is an ophthalmic emergency that can cause sudden loss of vision or even loss of light perception in patients, and it requires early diagnosis and treatment. With the increase in the incidence rate of coronary heart disease and the increasing number of invasive cardiac examinations and operations, cardiologists should improve their understanding of ocular complications of intravascular ultrasound. During intravascular ultrasound examination and related procedures, attention should be paid to changes in the patient's visual quality to alert cardiologists to possible retinal artery occlusion caused by intravascular ultrasound examination and other procedures. Preventing the occurrence of this complication is particularly important; therefore, the ultrasound catheter should be gently inserted and removed from the coronary artery during intravascular ultrasound examination to prevent embolism caused by intimal or plaque detachment.

## Data Availability

The original contributions presented in the study are included in the article/Supplementary Material, further inquiries can be directed to the corresponding author.
